# The Emergence and Future of Public Health Data Science

**DOI:** 10.3389/phrs.2021.1604023

**Published:** 2021-04-26

**Authors:** Jeff Goldsmith, Yifei Sun, Linda P. Fried, Jeannette Wing, Gary W. Miller, Kiros Berhane

**Affiliations:** ^1^ Department of Biostatistics, Columbia University Mailman School of Public Health, New York, NY, United States; ^2^ Columbia University Mailman School of Public Health, New York, NY, United States; ^3^ Data Science Institute, Columbia University, New York, NY, United States; ^4^ Department of Environmental Health Sciences, Columbia University Mailman School of Public Health, New York, NY, United States

**Keywords:** machine learning, big data, computational methods, ethics, reproducibility, interdisciplinary science

## Abstract

Data science is a newly‐formed and, as yet, loosely‐defined discipline that has nonetheless emerged as a critical component of successful scientific research. We seek to provide an understanding of the term “data science,” particularly as it relates to public health; to identify ways that data science methods can strengthen public health research; to propose ways to strengthen education for public health data science; and to discuss issues in data science that may benefit from a public health perspective.

## Background

Although the major components of data science have existed for many years, the term has rapidly grown in prominence in the last decade. This reflects the confluence of several important trends in science, including the prevalence of big data, the development of computational approaches to analysis, and recognized need for reproducibility in research. Meanwhile, public health has always emphasized rigorous study design and data collection, appropriate analysis methods, interdisciplinary research, and the ethical use of data. The emergence of public health data science reflects a dialog between these disciplines and promises transformative, lasting innovations in the use of data to advance population health.

In January 2020, the Mailman School of Public Health at Columbia University hosted the inaugural Data Science in Public Health Summit to examine the issues described in this commentary. With participants from over 60 schools and programs in public health, the Summit provided a forum for discussion and debate about the role of data science in the future of public health. In her opening remarks, Dean Linda Fried of the Mailman School reiterated the constant mission of public health–“to use science to raise the floor and the ceiling of health for everyone”–while recognizing the shifting landscape and dynamic challenges we face. A keynote address focused on innovative uses of data science methods to advance health, while a series of panels featuring leading experts discussed key research, educational, and ethical issues surrounding data science within the field of public health. The Summit was recorded, and all presentations and panel discussions can be viewed at https://tinyurl.com/yavhmlcf.

This commentary is contains our views on the present and future of public health data science. These views were shaped by the discussions at the Summit, but may not reflect the opinions of all Summit participants.

## Defining Public Health Data Science

In less than a decade, “data science” has transformed from a niche term describing a growing phenomenon to a broad concept invoked in almost every setting. Universities and research institutions have departments, centers, or institutes focusing on data science, either as a stand-alone discipline or a field spanning many traditional domains. Data science is becoming a popular undergraduate major, and graduate programs in the area have expanded rapidly to meet demand. Bootcamps, short courses, and certificate programs—in person and online, and through academic and non-academic platforms—have proliferated outside of traditional degree programs. Data scientists (identified by virtue of their formal training or demonstrated skillsets) are among the most sought-after members in the workforce, and the need for data scientists is expected to grow rapidly in coming years [1].

Despite the sudden prominence of data science [2], there continues to be debate over how the field should be defined. There are competing, and sometimes conflicting, perspectives; typically these include a combination of proficiency in computer science, grounding in statistics, and understanding of the relevant substantive domain, but views differ in the relative importance given to each [3]. In some instances, it has been argued that “data science” simply rebrands existing fields like statistics or computer science. Because of this indefinition, even researchers with clear quantitative expertise can be hesitant to present themselves as “data scientists”: doing so may seem like claiming mastery of an amorphous and constantly—changing collection of skills.

Our view is that data science has gained traction as an overarching term due to the convergence of several trends over the last decade or more. These trends are: increased data availability and complexity, which can result in “big data” settings; development of computational methods, especially those for prediction; advances in computational infrastructure, such as cloud computing and GPU clusters, that enable the processing of massive datasets; growing concerns about scientific rigor and the reproducibility of research findings; and a recognition that new advances will result from interdisciplinary research and collaboration. These trends are not unique to data science, but their integration and consolidation under a single term, however broad, reflects an understanding of their interconnectedness and is a real shift in the scientific landscape. Data science is better understood as a new conceptual perspective on scientific work than as a collection of specific skills or as a single discipline focused on prediction methods, and may result in a transformational change in the conduct of scientific research. It will fall to public health researchers and practitioners to shape and translate this perspective in the service of advancing population health.

Against this backdrop, we propose a definition to frame our discussion:

“Public health data science is the study of formulating and rigorously answering questions in order to advance health and well-being using a data-centric process that emphasizes clarity, reproducibility, effective communication, and ethical practices.”

This includes elements of hypothesis generation; study design; data collection, data storage, manipulation, processing; methods development and application for appropriate analysis; dissemination; and translation. Our definition draws on recognizable elements of existing disciplines within and outside of public health, and includes the specific goal of protecting and promoting health. This definition is accompanied by a connotation, so that “data science” implies a perspective that is shaped by the emergence of big data, prediction algorithms, computational approaches, reproducibility, and interdisciplinary work. This connotation, as much as any specific definition, helps explain the sudden valence of data science.

Public health is well situated not simply to react to the emergence of data science, but to lead in the ongoing evolution of this dynamic new field. Data have been the foundation of public health’s mission: to understand the burden of disease, disability and injury and the opportunity to improve health across the full life course, to recognize solutions to disparities, to infer causal mechanisms, and to provide evidence for the impact of interventions. Public health researchers are trained to think critically about the appropriateness of a study’s design to evaluate scientific hypotheses, and to interrogate the measurement and sampling processes that produce observations. Public health research is inherently interdisciplinary and collaborative, drawing from quantitative and qualitative expertise across domains to effect change in the health of populations. Crucially, public health is concerned with the ethical questions that surround data and prediction algorithms, and the impact these can have on exacerbating disparities in health outcomes. These long-standing public health competencies are clearly relevant to the future of data science.

## Data Science in Public Health Research and Practice

### Innovative Data Science Research Methods can Extend Public Health’s Reach

Data relevant to public health has grown in scale and complexity, and will continue to do so for the foreseeable future. Now-common observations on individuals that involve large quantities of data already include genomic and other information available through biosamples; exposure to mixtures of environmental pollutants; lifestyle behaviors measured continuously through wearable devices; detailed health care histories from electronic records; and the social media, search queries, digital records of on- and off-line transactions, and similar elements that make up an individual’s digital footprint. These data sources have emerged in parallel to the development of new analytical strategies and capabilities, including statistical or machine learning methods, prediction algorithms, and deep neural networks. Rich data and complex methods promise to reshape the questions public health researchers can ask and the ways in which those questions can be answered. To capitalize on this potential, it is necessary to synergistically combine data science approaches with the public health science perspectives.

There is an apparent conflict between the need for interpretable models in public health and the “black box” approaches often associated with methods in data science. In the long term, “explainable AI,” or artificial intelligence methods that yield interpretable predictions, may bridge this divide; development in this direction is underway [4]. More immediately, however, we see an opportunity to adapt new approaches to existing public health problems. For example, targeted maximum likelihood estimation provides estimates of causal effects using data from observational studies by building on ensemble prediction methods [5], and automated variable selection has been used to identify the predictors most associated with health outcomes from a large collection of features [6]. We also argue that the combination of computational methods and big data has increased the ability to generate new hypotheses by discovering patterns hidden by noise, scale, and complexity. Indeed, data-driven approaches to identifying important subgroups, using techniques like clustering, principal components analysis, and t-distributed stochastic neighbor embedding (t-SNE), are common even in the absence of statistical inference or clear interpretations. By understanding when and how cutting-edge approaches can be used, public health scientists will ensure that rich data are being used to their fullest potential.

Meanwhile, we recognize prediction accuracy as an important goal in itself. Identifying signals from massive and dynamic data, such as those at risk of death by suicide using data from social media posts and other sources, for example, would provide an avenue for timely intervention even without an interpretable model. Overall, through a focus on prediction accuracy, precision public health may seek “the right intervention for the right population at the right time” to improve health, in contrast to precision medicine’s patient- and treatment-centered outlook. In this framework, identifying optimal interventions and the right target population can be more important than interpreting model results. Initiatives like the NIH’s “All of United States,” the United Kingdom Biobank, and other resources that make complex data available at large scale, may make predictive algorithms attractive or even necessary analytic approaches to facilitate precision public health research [7, 8]. Prediction accuracy is complementary to interpretability: it may not be the highest priority in many settings, but should be understood nonetheless.

Clearly, data science for public health will rely on interdisciplinary teams to make advances–no single researcher, or even a research team comprised of members of a single discipline, will have the requisite breadth of expertise needed to solve problems in this environment. Successful teams will be well-versed in the behavioral, social, or biological determinants of the health outcome of interest; understand the complex systems that describe the determinants and outcomes; recognize the potential for opaque methods to propagate bias inherent in underlying data; and rely on quantitative experts to identify and implement appropriate analytic strategies. Increasingly, teams will integrate expertise in bioinformatics, computer science, engineering, and other quantitative domains that have been, to date, infrequent partners in public health research. As a consequence, institutions that adopt incentives to promote team science and actively seek to bridge silos of expertise will be leaders in public health data science; external groups, particularly those that fund research, should encourage this through initiatives that reward interdisciplinary work.

### Data Science Tools will Improve Current Public Health Practice

The popularity of advanced computational methods and prediction algorithms doesn’t mean the end of long-standing methods in public health research: odds ratios are in no danger of becoming obsolete. Data science emerged during a time of growing concern regarding the “reproducibility crisis” in science, and as a partial response emphasizes the adoption of tools for data analysis, project management, and collaboration that encourage transparent and reproducible research [9, 10]. These tools are code—and computer-centric, and are as relevant to “small data” studies that use traditional analytic techniques as they are to big data settings.

As anyone who has worked with data knows, there are many steps and a good deal of work between data collection and obtaining evidence from those data. “Data wrangling” involves importing, restructuring, cleaning, and otherwise organizing data for analysis. Exploratory data analysis can include computation of numeric summaries, visualization, and initial evaluation of hypotheses. Formal analysis of (ideally pre-specified) scientific hypotheses, the results of which are summarized and disseminated, follows these initial steps. This process is often non-linear and iterative: underlying data might be updated, for example, necessitating re-analysis. In practice, working with data can be time-consuming, ad-hoc, and even undervalued.

There is an emerging consensus that the processes supporting data analysis also require meritorious science themselves. Approaching data analytic work in a way that is deliberate, coherent, and consistent across projects has several benefits. A shared definition for “tidy data” allows researchers to focus on producing evidence by reducing the cognitive demand required to understand the structure of individual datasets. Using a common analysis workflow for all projects facilitates collaboration across members of a research team. Clear project organization, including raw data, cleaned data, code, and output, as well as tracked versions of these, can help promote reproducibility through transparency and structure. “Good enough practices” for working with software and data formalize this perspective and are accessible to the vast majority of public health researchers and analysts [11].

These strategies have become an understood element of doing data science in practice, and don’t depend on the use of specific software. However, some computational environments foster reproducibility directly rather than relying on proactive users. As an example, the R packages collectively known as the “tidyverse” implement tools designed around a shared philosophy for data structures, and are intended to focus researchers on extracting evidence rather than wrestling with datasets [12]. Meanwhile, the RStudio integrated development environment facilitates project organization, data analysis, reproducible reporting, and version control; this organization has also taken steps to foster a supportive and inclusive user community. These tools for data science, and similar infrastructure in other statistical programming languages, are successful when they make transparency and reproducibility the default behavior.

Adopting the tools of data science can strengthen the reproducibility of public health science, although we caution that these are not a panacea. By themselves, these tools don’t ensure appropriate study designs for the creation of datasets, interrogate potential biases in data sources, or assess the biological plausibility or public health relevance of findings. Just as public health research can be strengthened through the thoughtful adoption of data science practices, we believe data science will be improved by incorporating fundamental public health perspectives.

## Training Public Health Data Scientists

Public health is, of course, the most important component of public health data science, and training will continue to emphasize the time-proven and science-driven fundamental principles that underlie public health reasoning. Proficiency in the core competencies of epidemiology, biostatistics, and other disciplines that support public health will ensure that public health data scientists are able to understand the burden of disease and determinants of health; to think critically about study design, sampling, and measurement; and to quantify the evidence that data provides in support of hypotheses.

The distinct areas of data science described in the previous section—cutting‐edge methods in statistical learning and computer science and the computational infrastructure that underlies data science work—suggest changes to existing training programs in public health. First, traditional analysis methods should be complemented by instruction in more recent computational techniques, and these should be integrated into other core public health disciplines. Second, practices for transparency and reproducibility in data analysis should be taught explicitly and expected in all data-centric training components; many students will need at least basic proficiency in a programming language like R or Python. Supporting these, project-based learning has several benefits in general; in the context of data science, this approach can encourage a collaborative, interdisciplinary perspective.

Learners at different stages or with different backgrounds will have unique objectives for training in public health data science. Introductory courses targeting undergraduates, MPH students, and others with similar needs should focus on drawing distinctions between statistical methods used to make inferences and prediction-oriented computational tools, and understanding when each is appropriate; introducing concepts for reproducibility in research; and communicating effectively with quantitative experts. Others may need more explicit technical training in modern methods or in implementing those methods in novel settings, and could get this training from traditional coursework or from bootcamps focusing on particular topics. Because data science is relatively new, options for continuing education emphasizing practical skills may appeal to members of the public health workforce. In all of these domains, forming partnerships with computer scientists, engineers, and other disciplines with strength in data science will improve training in public health data science.

## Avenues for Public Health Contributions to Data Science

Data science is a developing field that draws on, and attempts to formalize, themes that are established components of quantitative research. Meanwhile, public health has always relied on a rigorous understanding of study design, data collection, and sources of bias; the principled use of quantitative methods; expertise in the content areas of health that can guide theory formation and testing; an interdisciplinary and translational approach; and a commitment to the ethical and responsible use of data. These strengths are as relevant now as ever, and offer clear directions for public health leadership in data science.

### Public Health Perspectives are Broadly Relevant

There is an inherent idealism among public health researchers and practitioners—members of this community have chosen to work in a field that is dedicated to improving the health and lives of populations—and that idealism is present in every analysis, study, and intervention. This is increasingly mirrored by a commitment to effecting positive change in the data science community. The Data Science Institute at Columbia University, for example, emphasizes the use of “Data for Good” to reflect the Institute’s dedication to benefitting society through responsible data science approaches. Other groups share similar goals, and members of the data science community are focused on medicine, health, inequality, and justice.

We are supportive of this trend, and look forward to more fully integrating a public health perspective into data science. Research on human subjects imposes clear responsibilities that the community takes very seriously, including respect for persons, beneficence, and justice, among others. From a practical perspective, public health researchers are required to learn from data that can be messy and imperfect, and have been trained to question whether an observation is a valid measure for a construct of interest, if selection biases create a mismatch between the sample and target population, and if the study design allows for rigorous statements about the hypotheses of interest. A shift toward complex data and analytic approaches makes these considerations more critical than ever; indeed, we worry that there are risks associated with the use of data science to address health-related questions in the absence of these considerations.

Training in data science should include a public health component, emphasizing core competencies in epidemiology, biostatistics, and ethics, and interdisciplinary data science teams should include experts in public health. This is critical for data scientists and teams interested in health, but relevant beyond this domain. Indeed, a public health perspective is important in settings that are not obviously health-related: the potential for bias in deep learning algorithms used to screen job applicants; the concern that social media recommendation systems can lead to information silos; and the possible use of facial recognition and other biometric surveillance mechanisms by law enforcement. In these areas and others, data scientists should understand the processes that give rise to available data, how those processes can shape conclusions, and what data uses might lead to just outcomes. We believe a close collaboration between data science and public health will advance this perspective.

### Public Health can and Should Lead in Ethics for Data Science

The public health community can draw on experience in human subjects research to lead in defining and promoting ethical and responsible data science in a framework of unintended consequences that could possibly harm health and wellbeing.

“Big data” create novel opportunities for research and innovation, but pose commensurate ethical risks and challenges [13]. Large observational datasets gathered through passive surveillance or scraped from online sources raise questions about representation, consent, privacy, and the responsibilities researchers have to subjects. Data of the scale required by many prediction algorithms are often collected through mechanisms that can introduce bias: electronic health records rely on engagement with the health care system, and persistent racial disparities in cancer outcomes may be linked (or missed due) to a lack of diversity in research subjects. By virtue of this scale, such data can give a misleading perception of representativeness and the illusion of fairness that, if unexamined, can lead to erroneous conclusions and faulty policies. The questions that public health researchers are trained to ask about key components in the data life cycle are foundational to the ethics of data and data science [14].

Ethical challenges persist or are introduced after data are available. Algorithms trained on imperfect data can be misleading whether the data are small or big, and will perpetuate biases inherent in data unless actively prevented from doing so. The identification of causes, rather than associations, relies on subject-area expertise and appropriate methods. Using results from a sample to understand effects in a population requires understanding of selection and sampling. Here, too, public health leadership is relevant. In interdisciplinary teams, advocating a healthy skepticism about the quality of data and identifying potential sources of bias is crucial, as is proactively conducting checks on the validity of analysis results. The skills necessary for this role should be updated for modern data and approaches, and to the extent possible formalized in steps that can be conducted routinely.

Ensuring complex approaches are used for ethical purposes is, we believe, one avenue through which public health researchers will have an impact on data science. We also argue that asking whether these approaches are inherently ethical is a challenge the field must address. It may not be immediately obvious to question the ethics of algorithms, but several such questions exist. How frequently does a method have unintended negative outcomes? Are extrapolations that are unsupported by data masked by model complexity? Does an algorithm rely on data that were not ethically obtained, or produce data without subjects’ consent? Does the algorithm, through the selection of a criterion to be optimized, contain implicit biases? The answers to these questions may illustrate when and how (or whether) a method should be used, and what safeguards are necessary to ensure just outcomes.

## Outlook and Conclusion

We have defined and discussed public health data science, but have not precisely located this field with respect to public health or public health’s constituent disciplines. This reflects current reality–individuals who identify as public health data scientists often do so as a secondary discipline, with primary expertise in epidemiology, biostatistics, health policy, environmental health, or another area. It’s possible that this will remain true, but the present state may also be attributable to institutional organization around traditional fields or the ways that organization has shaped training in the past. Over time, public health data science may emerge as a primary domain among those for whom the implied perspective resonates, suggesting a need for institutional flexibility or reorganization. In any case, we want to be clear that public health data science does not simply rebrand an existing discipline like epidemiology or biostatistics; this view is flawed in a way that is analogous to dismissals of data science as a new term for computer science.

Whether the public health data science persists in a loose, interdisciplinary form or solidifies as a distinct field is not something we can predict, and will depend both on how existing disciplines define themselves moving forward and the ways data science itself evolves over time. While cannot predict the future, we do look forward to an ongoing, mutually transformative partnership between public health and data science that will strengthen both disciplines and the improve ability to extract from data the actionable insights that advance health.
